# Comparison of Conventional and Microwave Treatment on Soymilk for Inactivation of Trypsin Inhibitors and In Vitro Protein Digestibility

**DOI:** 10.3390/foods7010006

**Published:** 2018-01-08

**Authors:** Brinda Harish Vagadia, Sai Kranthi Vanga, Ashutosh Singh, Yvan Gariepy, Vijaya Raghavan

**Affiliations:** 1 Department of Bioresource Engineering, Faculty of Agriculture and Environmental Studies, McGill University, Sainte-Anne-de-Bellevue, QC H9X 3V9, Canada; brinda.vagadia@mail.mcgill.ca (B.H.V.); yvan.gariepy@mcgill.ca (Y.G.); vijaya.raghavan@mcgill.ca (V.R.); 2 School of Engineering, University of Guelph, Guelph, ON N1G 2W1, Canada; asingh47@uoguelph.ca

**Keywords:** soymilk, microwave processing, thermal processing, trypsin inhibitors, response surface methodology

## Abstract

Soymilk is lower in calories compared to cow’s milk, since it is derived from a plant source (no cholesterol) and is an excellent source of protein. Despite the beneficial factors, soymilk is considered as one of the most controversial foods in the world. It contains serine protease inhibitors which lower its nutritional value and digestibility. Processing techniques for the elimination of trypsin inhibitors and lipoxygenase, which have shorter processing time and lower production costs are required for the large-scale manufacturing of soymilk. In this study, the suitable conditions of time and temperature are optimized during microwave processing to obtain soymilk with maximum digestibility with inactivation of trypsin inhibitors, in comparison to the conventional thermal treatment. The microwave processing conditions at a frequency of 2.45 GHz and temperatures of 70 °C, 85 °C and 100 °C for 2, 5 and 8 min were investigated and were compared to conventional thermal treatments at the same temperature for 10, 20 and 30 min. Response surface methodology is used to design and optimize the experimental conditions. Thermal processing was able to increase digestibility by 7% (microwave) and 11% (conventional) compared to control, while trypsin inhibitor activity reduced to 1% in microwave processing and 3% in conventional thermal treatment when compared to 10% in raw soybean.

## 1. Introduction

Soymilk is a high protein liquid with considerable amounts of carbohydrates, fats, essential vitamins and mineral, generally produced by grinding soaked soybeans in excess water, which is then filtered to separate out the milk from solids and fiber. It is a stable oil in water emulsion, where the continuous phase is formed by dispersed soybean protein. Soymilk is composed of 94% water, 3% protein, 1.5% fat and 1.5% of carbohydrates. It also contains 7.36 and 0.33 mg/100 mL of riboflavin and thiamin, respectively, a composition similar to cow’s milk but with little-saturated fat and no cholesterol [1,2,3]. The nutritional profile of soymilk and cow’s milk (3.25% milkfat) are summarized in Table 1 [4]. 

In recent years, the consumption of soymilk has increased, especially among consumers who are lactose intolerant, vegetarian, vegan and/or seeking healthy diets. It is also considered safe for children with galactosemia [5], as galactose is absent in soymilk. In developing countries, soymilk is used as a low-cost substitute for cow’s milk in many food preparations [6,7]. This increase in consumption of soymilk can also be attributed to the presence of high-quality protein and low-fat content [2]. In 1999, the U.S. Food and Drug Administration approved the health claim for soy protein, which states that its consumption may reduce the risk of heart diseases by lowering the levels of low-density lipoproteins adding to its acceptance by a wide variety of consumers [8]. Several researchers have also associated the consumption of soy products to reduced risks of coronary heart diseases, atherosclerosis, type 2 diabetes, colorectal cancer, breast cancer and prostate cancer [9,10,11].

Despite all the beneficial factors, the nutritional value of soy milk is reduced by the presence of a variety of anti-nutritional factors such as Kunitz trypsin inhibitors (KTI), Bowman-Birk inhibitors (BBI) and lipoxygenase (LOX). Soybean contains the highest amount of protease inhibitors that accounts for two to six percent of whole soybean protein [12]. These proteases (KTI and BBI) inhibit the enzymatic activity of trypsin and chymotrypsin, the primary digestive enzymes responsible for reducing the proteins into dipeptides and tripeptides. The KTI has a molecular weight of 20 kDa with two disulfide bridges and exhibits specificity to inhibit trypsin. BBI has a molecular weight of eight kDa with seven disulfide bonds and exhibits specificity to inhibit chymotrypsin and trypsin [13,14]. Rouhana et al. reported 60% of soymilk trypsin inhibition activity (TIA) was from KTI [15]. High levels of active KTI have been shown to reduce protein digestibility and cause pancreatic carcinogenesis upon consumption [16]. In animals, protease inhibitors have been associated with growth suppression and pancreatic hypertrophy, emphasizing the need for identification and development of effective techniques to reduce their presence in soy products [17,18,19]. Soybean trypsin inhibitors are heat stable and require a long processing time. According to Yuan et al., TIA values decreased to 13% of the original raw soymilk TIA values when processed by the traditional thermal treatment (heating at 100 °C for 20 min) [20]. However, the long processing time may affect the other nutritional properties of soy products and hence should be avoided [20,21]. At the same time, 100% inactivation of trypsin inhibitors (TI) causes overheating and damages the proteins by destroying lysine, tryptophan and cysteine in soymilk. Thus, extended periods of thermal treatment inactivate TI effectively, but it denatures essential soybean proteins resulting in amino acid degradation, browning reaction and other deteriorative reactions [20,21]. The flavor, color, and vitamin content are also affected depending on the type of heat treatment used [21,22]. Hence, processing plays an essential role in the sensory appeal and nutritive value of soybean and soy products including soymilk. The various factors to be considered for a good quality soymilk during processing are yield, nutritional quality, anti-nutritional profile, color attributes, particle size, texture profile and organoleptic quality [14,23]. 

Moreover, there are still questions concerning the ideal processing conditions to produce commercially sterile soymilk with minimum nutrient degradation. Manufacturing techniques are required that have shorter processing time, are energy efficient (environment-friendly), have lower production costs and maintain the quality of soymilk [24]. Autoclaving, batch boiling and steam injection [25,26], Ultra-High Temperature (UHT) [27], High Temperature and Pressure combination [28], Ohmic heating [29], and High-Pressure Processing (HPP) [17] are processing methods that have been explored for inactivation of TI in soymilk.

Industrial scale dielectric heat treatment technology at 42 MHz (Radio frequency) and 2450 MHz (Microwave) were found to be effective against TI in soybean and these methods also improved the overall quality of the protein. The processing time required to reach safe levels of TI inactivation is less in microwave treatment when compared to conventional methods for soybeans [30]. Studies by Barac and his team showed that the TI levels were reduced to 13% of the initial value in soybean during microwave roasting at 2.45 GHz for two min [31]. In a study conducted by Yoshida et al., the inactivation of the anti-nutritional factors to safe limits of soaked soybean at 2.45 GHz requires only four min [32]. In comparison, the conventional batch boiling process takes 15 min at 100 °C to inactivate the levels of TI to 20% [15]. To the best of our knowledge, no studies have been done on the inactivation of soybean trypsin inhibitor in soymilk using microwave processing despite the upsides of using this dielectric processing technique. This can be regarded as a potential alternative to existing conventional processing methods in the food industry for inactivation of anti-nutritional factors.

This study reports the effect of microwave processing on the reduction of TIA in comparison to the conventional thermal processing of soymilk. In vitro Protein digestibility (IVPD) studies were also performed to assess the effects of microwave processing on its digestibility at different time and temperature conditions. Optimization of these processing techniques was performed using Response Surface Analysis.

## 2. Materials and Methods

Soybeans (*Glycine max*) was procured from Goliath, QC, Canada. Initial moisture content was found to be 10.1% on a wet basis. The moisture content was determined by AOAC official method for moisture content in soybean flour by hot air oven method. Soybean flour (5 g) was dried in an oven at 130 °C ± 3 °C for two hours, after which the weight became constant [33]. Fresh soymilk was prepared from fresh soybean before performing thermal and microwave processing.

### 2.1. Soymilk Preparation

Soybeans were washed, cleaned and soaked in distilled water in the ratio 1:10 (*w*:*v*) (bean:water) for 18 h at room temperature (25 °C) for complete hydration. The soymilk was prepared by wet grinding the hydrated soybeans along with water for three mins at high speed in a stainless-steel blender (Nutri Bullet, NutriBullet LLC, Pacoima, CA, USA). The slurry was filtered through a double layer of cheesecloth to separate out the solids from soymilk. Raw soymilk obtained had a pH of 6.5 [8,34].

### 2.2. Solvents and Reagents

All reagents and solvent used were of High-Performance Liquid Chromatography (HPLC) grade and were purchased from Fisher Scientific (Ottawa, ON, Canada). The enzymes used for in vitro Protein Digestibility (IVPD %) determination and trypsin inhibitor assay were purchased from Sigma Aldrich (Oakville, ON, Canada).

### 2.3. Conventional Thermal Treatment

For conventional thermal treatment, 30 mL of soymilk was placed in a water bath which was pre-set and maintained at the processing temperatures of 70 °C, 85 °C and 100 °C. The samples were treated for 10, 20 and 30 min in the water bath. All the experiments were conducted in triplicate. After cooling at room temperature, the samples were collected, stored overnight at 40 °C and later freeze-dried in a laboratory freeze-dryer (Gamma 1-16 LSC Freeze dryer, Martin Christ Gefriertrocknungsanlagen GmbH, Osterode am Harz, Germany) and stored in opaque air-tight containers at −20 °C until further analysis was conducted.

### 2.4. Microwave Processing

The microwave processing was conducted using the MiniWAVE digestion system (SCP Science, Baie-D’Urfe, QC, Canada) that operates at a frequency of 2.45 GHz at 1000 watts. The soymilk samples were heated in cylindrical quartz reactor vessels. The experiments were conducted at processing temperatures of 70 °C, 85 °C and 100 °C for 2, 5 and 8 min. The sample temperature was monitored using Infra-red (IR) sensors located on the sidewalls and displayed in real time on the controller screen during the run. The MiniWAVE system uses a single magnetron located below the floor of the chamber. After the treatment, the reactor vessels were cooled to room temperature gradually by the cooling unit of the microwave system. The samples were stored until further analysis in the same manner as that of conventionally treated samples.

### 2.5. Chemical Analysis

#### 2.5.1. In Vitro Protein Digestibility (Multi Enzyme Method)

The In-vitro Protein Digestibility (IVPD) of soybean protein was evaluated using the multi-enzyme method. The working protein suspension was prepared by dissolving samples to yield 312.5 mg of protein in 50 mL of distilled water, whose pH was adjusted to 8.0 using 0.1 N NaOH and 0.1 N HCl. A multi-enzyme mixture was prepared, containing 1.6 mg/mL trypsin, 3.6 mg/mL chymotrypsin, and 1.3 mg/mL peptidase and its pH was adjusted to 8.0. The mixture was placed in an ice-bath and continuously stirred [35,36,37,38]. Five milliliters of the multi-enzyme solution were added to the samples, which were maintained at 37 °C in a water bath for the digestion with continuous stirring. The pH was measured after 10 min of the digestion and IVPD was calculated using Equation (1) [37].

IVPD % = 210.46 − (18.10 × pH_10min_) (1)

#### 2.5.2. Trypsin Inhibitor Assay

In this study, the total Trypsin inhibitor assay was assessed using the procedure followed by Hamerstrand et al. [39,40] with some modifications. Freeze dried soy milk (0.5 g) was extracted with 50 mL of 0.01 M NaOH for three hours, with constant stirring at room temperature. The suspension was then allowed to stand for two hours at 4 °C. The supernatant from each sample was collected and diluted, such that 2 mL of the extract could produce 40–60% trypsin inhibitor activity. 

Trypsin (type 1× from bovine pancreas, Sigma Chemical Co.) was used as a standard. Diluted soymilk supernatant (1 mL) was pipetted into test tubes in triplicates containing 2 mL of trypsin solution (20 mg in 0.001 M HCl). The control sample (blank) consisted of diluted sample extract and distilled water. The tubes were preheated at 37 °C for 10 min and then, 5 mL of benzyl-DL-arginine-para-nitroanilide (BAPNA), pre-warmed to 37 °C, and was added to each of the tubes and vortexed. After incubating this mixture at 37 °C for 10 min, the reaction was stopped by adding 1 mL of acetic acid (30%). The samples were centrifuged at 3000 g for 10 min. The absorbance of the clear supernatant was measured using spectrophotometer at 410 nm [41,42]. TIA is calculated in terms pure trypsin/g sample as weighed (mg/g).

TIA = (2.632 × D × A_I_)/S (2)

where D is the dilution factor (factor by which the original soymilk sample was diluted to obtain an inhibition between 40% and 60% by 1 mL of the diluted extract), S is the sample weight and A_I_ is the change in absorbance due to trypsin inhibitor/mL diluted sample extracted.

### 2.6. Statistical Design and Analysis

In this study, a response surface methodology including the design of experiments, fitting of mathematical model and optimization of processing condition for soymilk samples was employed. The central composite design (CCD) with uniform precision was applied for two independent factors, namely temperature (*X*_1_) and time (*X*_2_), each at three levels (−1, 0, and +1) as shown in Table 2. The design used to plan experiments consisted of a total of 14 combinations with six central, four factorial and four axial points combinations as shown in Table 3. The responses: TIA and IVPD were recorded. JMP software version 11 (SAS Institute Inc., Cary, NC, USA) was used for the experimental design and analysis. The functional relationship between the factors (*X_i_*, *X_j_*, *X_k_*, etc.) and responses (*Y*) was unknown, hence a regression model (Equation (3)) was used to analyze the actual response surfaces [43,44,45].

(3) $$Y=\beta_{0}+\sum_{i=1}^{i=n}\beta_{i}X_{i}+\sum_{i=1}^{i=n}\beta_{ii}X_{i}^{2}+\sum_{i=1}^{i=n}\sum_{j=1}^{j=n}\beta_{ij}X_{i}X_{j}$$

where β_0_ is the constant coefficient, β*_i_* is the linear coefficient, β*_ii_* is the quadratic coefficient for main process parameters and β*_ij_* is the second order interaction coefficient of variables *i* and *j*, respectively. The statistical design was prepared taking the temperature in °C and time in min. Separate CCD was prepared for both conventional thermal processing and microwave processing method. F value and its significance, Lack of Fit (LOF), and the coefficient of determination (*R*^2^) were assessed and the ANOVA analysis of the predictive model with the corresponding significant terms were reported in Table 5, Table 6, Table 8, Table 9. The differences among the treatments were also detected using Duncan multiple-range test using the probability level 0.05 [46].

## 3. Results and Discussion

### 3.1. Optimization of Conditions for IVPD during Microwave and Conventional Processing

**Figure 1:**
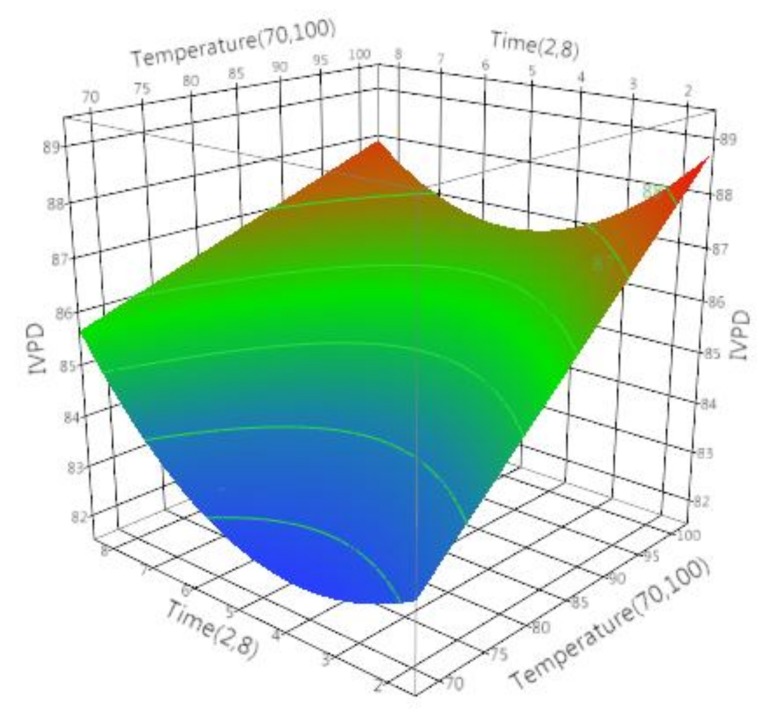
Effect of time (min) and temperature (°C) on in vitro protein digestibility (IVPD) of soymilk during microwave processing.

Legumes are known to have a lower protein digestibility, which is attributed to the presence of anti-nutritional factors [14,47]. On average, IVPD of microwave processed and conventionally treated soymilk samples was 85 ± 1.5% and 88 ± 2.0%, respectively (shown in Table 4). The nutritional quality of soybean protein cannot be determined by its amino acid composition alone, but its digestibility in the small intestine and determining the bioavailability should also be considered. Our investigations of the results for microwave and conventional processing of soymilk indicated that both the independent factors; temperature (temp) and time (*t*) significantly (*p* ≤ 0.05) affected the IVPD of soy proteins (Table 5 and Table 6). Overall, the regression model developed after ANOVA analysis was significant (*p* < 0.05) for both the treatments with insignificant lack of fit (*p* > 0.05). For both treatments, it can be interpreted that, by increasing the treatment temperature and time, an increase in IVPD was observed. In the case of microwave processing, from the values of parameter estimates or regression coefficients it was concluded that the most influential factor affecting the IVPD is the temperature with the highest regression coefficient of 1.694, followed by a square term of time, 1.250, a linear term of time, 0.3519, and lastly the interactive term of temperature and time with −0.620 regression coefficient. The negative regression coefficient for the interactive term (temp × time) suggested that as the microwave processing time was increased at any processing temperature a slight decrease in IVPD was observed till the time reached approximately 5 min and later the IVPD increased (Figure 1). This observation led to a conclusion that can be related to the changes in the conformation of proteins under the influence of oscillating electric field of microwave (2.4 GHz). As the sample was subjected to microwave processing a change in the confirmation of soymilk protein would reduce its susceptibility to the digestive enzymes but as the processing time increased the protein would denature and digestion would proceed as desired. This conclusion is based on previous observations made by the researchers though molecular modelling studies conducted to evaluate the effect of oscillating and static electric fields on various food proteins including peanut and soybean hydrophobic proteins [48,49,50]. Similar studies were conducted to understand the structure and digestibility in other legumes such as dry beans (*Phaseolus vulgaris*) and green peas (*Pisum sativum*) [51], sorghum (*Sorghum bicolor*) and maize (*Zea mays*) [52,53].

The predictive quadratic model (Equation (4)) generated for microwave processing of soymilk was significant (*p* < 0.0001) with *R*^2^ value of 0.83 and insignificant lack of fit.

(4) $$\begin{array}{l} {\mathrm{IVPD}}_{\mathrm{microwave}}=84.683+1.694\left[\frac{\left(\mathrm{Temperature}-85\right)}{15}\right]+0.351\left[\frac{\left(\mathrm{Time}-5\right)}{3}\right]+\\ \left[\frac{\left(\mathrm{Temperature}-85\right)}{15}\times \left[\frac{\left(\mathrm{Time}-5\right)}{3}\right]\times -0.620\right]+\left[\left[\frac{\left(\mathrm{Time}-5\right)}{3}\right]\times \left[\frac{\left(\mathrm{Time}-5\right)}{3}\right]\times 1.250\right] \end{array}$$

**Figure 2:**
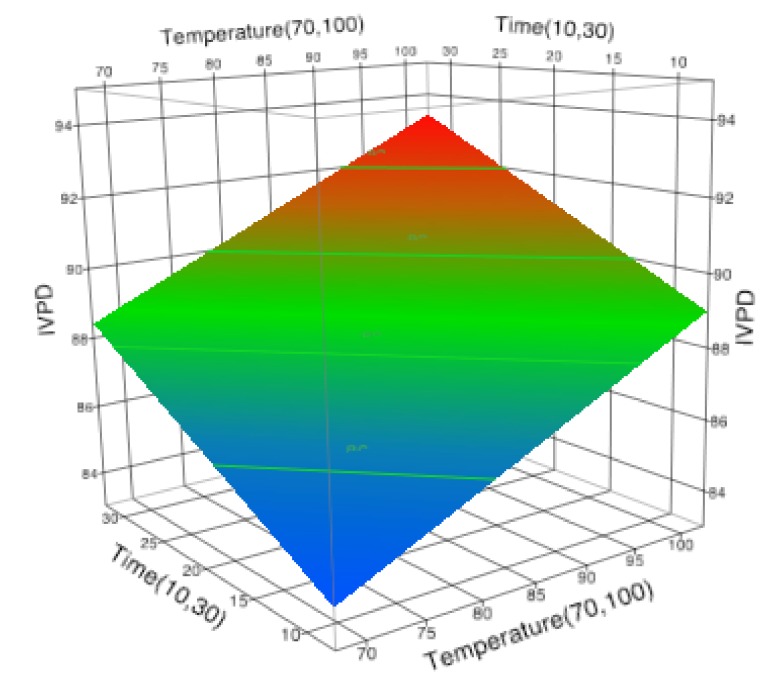
Effect of time (min) and temperature (°C) on in vitro protein digestibility (IVPD) of soymilk during conventional thermal treatment.

As in the case of conventional processing, it was observed that the most influential factor was the temperature with a regression coefficient of 2.127 followed by time with 1.765 regression coefficient. None of the other model parameters including the cross terms and square terms were significant leading to a linear regression model (Equation (5)) with *R*^2^ of 0.80. Several researchers have suggested that the treatment temperature is the key determinant of food protein digestibility [54,55]. A similar linear relationship between treatment time and temperature were observed by Wallace et al. (1971) in their study on the effect of different heat processing conditions on the TIA and the IVPD of various soymilk preparation (Figure 2). They reported that digestibility of proteins increased with an increase of the heat treatment and it also coincided with a decrease in the TIA [26]. Our study showed similar results, maximum digestibility of soymilk proteins occurred at 100 °C for 30 min of conventional processing. Other studies establishing the relationship of increase in digestibility due to a decrease in anti-nutritional factors were seen in rice [56], cowpea [57], chickpeas [58], moth beans [59], and common beans [60].

(5) $$\mathrm{IVP}D_{\mathrm{connventional}}=88.731+2.127\left[\frac{\left(\mathrm{Temperature}-85\right)}{15}\right]+1.765\left[\frac{\left(\mathrm{Time}-20\right)}{10}\right]$$

### 3.2. Optimization of Conditions for TIA during Microwave and Conventional Processing

**Figure 3:**
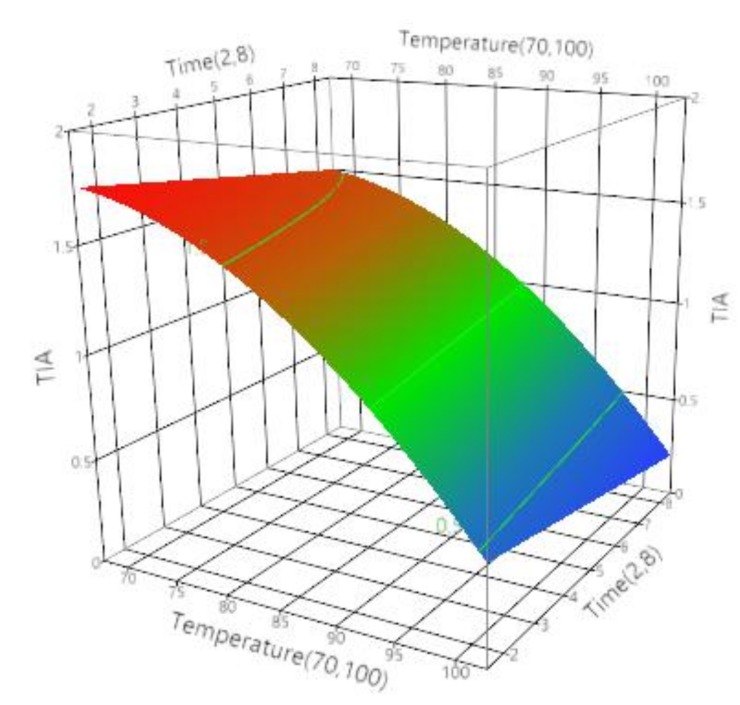
Effect of time (min) and temperature (°C) on trypsin inhibitor activity (TIA) of soymilk during microwave processing.

Trypsin inhibitor activity (TIA) governs the nutritional value of soymilk protein [61,62]. The average values for TIA during microwave and conventional processing is mentioned in Table 7. It has been reported by several researchers that overheating for complete removal of TIA reduces the overall nutritive value of soybeans [63]. Hence, a precise, controlled thermal process or a novel process is required for preparation of soymilk with maximum nutritive value. In this study analysis of the effect of microwave and conventional processing of soymilk on TIA revealed that both temperature and time play a significant (*p* < 0.05) role in determining it. Table 8 presents the ANOVA analysis for the effect of temperature and time on TIA for microwave processing. From the table, we can observe that regression model developed was significant with (*p* < 0.0001), insignificant lack of fit (*p* > 0.05) and *R*^2^ of 0.91 and that the independent factors temperature, its square term and time played a significant role. The regression coefficient analysis also supported the aforementioned observation, where temperature with regression coefficient estimate of −0.558, followed by its cross term with regression coefficient estimate of −0.192 and lastly time with a regression coefficient of −0.108 suggested that TIA decreases with increase in temperature and time (Figure 3), but overall presence of the significant temperature square term suggested temperatures significant influence on TIA. According to Rajko et al., inactivation of trypsin inhibitors requires more absorbed heat energy for longer processing time [64]. Similar experimental results were obtained by Alajaji and El-Adawy, during the microwave oven cooking of chickpea on high temperature for 15 min [58]. According to the studies by Oliveria and Haghighi, reduction in TIA was more pronounced in samples at higher temperatures as was expected because soybean TI loses activity irreversibly in the temperature range 80–110 °C [65]. In addition, studies by Esaka et al., TIA was not detectable after microwave heating at 120 °C for 5 min in case of winged bean seeds [66]. The predictive quadratic equation for effect of microwave processing on TIA was obtained as (Equation (6)).

(6) $$\begin{array}{l} {\mathrm{TIA}}_{\mathrm{Microwave}}=1.245+\left(-0.558\left[\frac{\left(\mathrm{Temperature}-85\right)}{15}\right]\right)+\left(-0.108\left[\frac{\left(\mathrm{Time}-5\right)}{3}\right]\right)+\\ \left(\left[\frac{\left(\mathrm{Temperature}-85\right)}{15}\right]\times \left(\left[\frac{\left(\mathrm{Temperature}-85\right)}{15}\right]\times -0.191\right)\right) \end{array}$$

**Figure 4:**
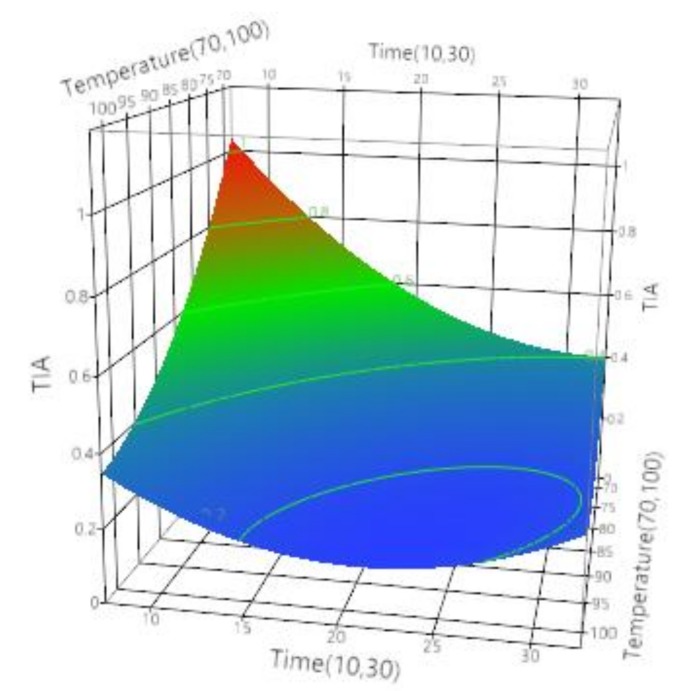
Effect of time (min) and temperature (°C) on trypsin inhibitor activity (TIA) of soymilk during conventional thermal treatment.

For the conventional process of soymilk and its effect on trypsin inhibitor activity it was observed that all the independent factors and its quadratic or cross terms significantly affected it (*p* < 0.05). As seen in Table 9, the predictive model generated was significant (*p* < 0.0001) with insignificant lack of fit and *R*^2^ of 0.77. The response surface graph presented in Figure 4 shows that highest TIA was obtained at lowest temperature for shortest processing time. As the processing time was increased, the TIA decreased at any given temperature. This observation can also be confirmed with analysis of regression coefficients of process parameters, which suggested that the most influential parameter was a temperature with regression coefficient estimate of −0.173, followed by time with −0.138, and their cross term with 0.099 and lastly with a square term of time, 0.102, and temperature, 0.099. Similar results were observed in lentil, chickpea and pea flours that were thermally processed by boiling in a water bath at 90 °C for 20 min, which significantly reduced the levels of trypsin inhibitors [67]. According to the results of Osman et al., hydrothermal treatment of Tepary bean extract at 100 °C for 60 min completely inactivated the TI [68]. Similar results were observed by Andrade et al., recommending a dry heat of 200 °C for 20 min for soy flour samples, showing temperature plays an essential role in inactivation of TI [69]. The predictive quadratic regression model generated for conventional processing is presented by Equation (7).

(7) $$\begin{array}{l} {\mathrm{TIA}}_{\mathrm{conventional}}=0.228+\left(-0.173\left[\frac{\left(\mathrm{Temperature}-85\right)}{15}\right]\right)+\left(-0.138\left[\frac{\left(\mathrm{Time}-20\right)}{10}\right]\right)+\\ \left(\left[\frac{\left(\mathrm{Temperature}-85\right)}{15}\right]\times \left[\frac{\left(\mathrm{Time}-20\right)}{10}\right]\times 0.099\right)+\left(\left[\frac{\left(\mathrm{Temperature}-85\right)}{15}\right]\times \left[\frac{\left(\mathrm{Temperature}-85\right)}{15}\right]\times 0.099\right)\\ +\left(\left[\frac{\left(\mathrm{Time}-20\right)}{10}\right]\times \left[\frac{\left(\mathrm{Time}-20\right)}{10}\right]\times 0.102\right) \end{array}$$

Thus, to obtain soymilk with maximum digestibility with inactivation of TI, microwave processing (2.45 GHz) at 100 °C for 8 min is recommended, in comparison to conventional processing at 100 °C for 30 min.

## 4. Conclusions

In this study, microwave processing (2450 MHz, 1000 Watts) at different conditions of temperature (70 °C, 85 °C and 100 °C) and time (2, 5 and 8 min) were applied to soymilk samples, in comparison to conventional thermal treatments at the same temperatures and time (10, 20 and 30 min). The IVPD increased with increase in time and temperature during microwave processing (100 °C for 8 min) and conventional processing (100 °C for 30 min) to 87% and 92%, respectively. This is higher compared to an initial digestibility of raw soymilk, which was estimated to be 80.5%. Similarly, TIA for conventional treatment (100 °C for 30 min) is 1% and for microwave processing (100 °C for 8 min) is 3% from an initial TIA of 10% of raw soymilk. Hence, microwave processing can be used as a potential alternative method of processing soymilk for increased digestibility and elimination of anti-nutritional factors.

## Figures and Tables

**Table 1:** Nutritional profile of soymilk and cow’s milk (unfortified).

Nutrients	Soymilk	Cow Milk
Water	85.61	88.13
Protein	2.26	3.15
Dietary fiber	0.4	0.0
Calcium	0.025	0.113
Carbohydrates	9.95	4.80
Sugars	7.86	5.05
Potassium	0.143	0.132
Cholesterol	0	10
Trans fatty acids	0	-

All the values reported are per 100 g. Report: 01077 and 16166, United States Department of Agriculture (USDA) Database.

**Table 2:** Central composite design for processing of soymilk with independent variables and their coded and actual values.

Process Parameters	Units	Coded Levels		
		−1	0	+1
Temperature	°C	70	85	100
Time (Microwave processing)	Min	2	5	8
Time (Conventional water bath)	Min	10	20	30

**Table 3:** Central composite design showing different combinations of temperature and time for processing of soymilk.

Experimental Run	Temperature (°C)	Time (min) Microwave Processing	Time (min) Conventional Processing
1	70	2	10
2	70	5	20
3	70	8	30
4	85	2	10
5	85	8	30
6	100	2	10
7	100	5	20
8	100	8	30
9–14	85	5	20

**Table 4:** Summarized statistics for in vitro protein digestibility of soymilk processing using process parameters according to central composite design.

	Microwave Processing	Conventional Processing
Range (% digestibility)	82–89	84–92
Average	85.2183	88
Standard deviation	±1.4586	±2.018

**Table 5:** ANOVA for effect of time (*t*) and temperature (temp) for in vitro protein digestibility for microwave processing.

Source	DF *	Sum of Squares	Mean Square	F Ratio	*p*-Value
Model	4	74.5361	18.6340	46.5061	<0.0001
Temperature (temp)	1	51.6754		128.9695	<0.0001
Time (*t*)	1	2.2296		5.5645	0.0237
Temp × *t*	1	4.5892		11.4537	0.0017
*t*^2^	1	16.0418		40.0366	<0.0001
Lack of fit	4	3.4025	0.8506	2.4575	0.0648
Error	37	14.8251	0.4007		
C. Total	41	89.3613			
Pure Error	33	11.4225	0.3461		
Total Error	37	14.8251			

* DF: degrees of freedom.

**Table 6:** ANOVA for effect of time (*t*) and temperature (temp) for in vitro protein digestibility for conventional processing.

Source	DF	Sum of Squares	Mean Square	F Ratio	*p*-Value
Model	2	137.5613	68.7807	79.9635	<0.0001
Temperature (temp)	1	81.4675		94.6810	<0.0001
Time (*t*)	1	56.0940		65.1919	<0.0001
Lack of fit	6	4.2183	0.7030	0.7908	0.5836
Error	39	33.5572	0.8604		
C. Total	41	171.1185			
Pure Error	33	29.3388	0.8891		
Total Error	39	33.5572			

**Table 7:** Summarized statistics for trypsin inhibition activity of soymilk processing using process parameters according to central composite design.

	Microwave Processing	Conventional Processing
Range (% inhibition)	3–18.8	1–9
Average	11.6	3.1
Standard deviation	±4.0333	±2.002

**Table 8:** ANOVA for effect of time (*t*) and temperature (temp) on trypsin inhibitor activity for microwave processing.

Source	DF	Sum of Squares	Mean Square	F Ratio	*p*-Value
Model	3	6.2115	2.0705	126.6718	<0.0001
Temperature (temp)	1	5.6224		343.9744	<0.0001
Time (*t*)	1	0.2112		12.9241	0.0009
Temp^2^	1	0.3778		23.1169	<0.0001
Lack of fit	5	0.0814	0.0163	0.9956	0.4356
Error	38	0.5871	0.0163		
C. Total	41	6.8326			
Pure Error	33	0.5397	0.0163		
Total Error	38	0.6211			

**Table 9:** ANOVA for effect of time (*t*) and temperature (temp) for trypsin inhibitor activity for conventional processing.

Source	DF	Sum of Squares	Mean Square	F Ratio	*p*-Value
Model	5	1.3021	0.2604	24.5188	<0.0001
Temperature (temp)	1	0.5408		50.9176	<0.0001
Time (*t*)	1	0.3472		32.6918	<0.0001
Temp^2^	1	0.1180		11.1108	0.0020
Temp × *t*	1	0.0835		7.8624	0.0081
*t* ^2^	1	0.0892		8.4001	0.0064
Lack of fit	3	0.2551	0.0850	22.0541	<0.0001
Error	36	0.3823	0.0106		
C. Total	41	1.6844			
Pure Error	33	0.1272	0.0040		
Total Error	36	0.3823			
